# Risk factors of preoperative Hirschsprung-associated enterocolitis

**DOI:** 10.1186/s12919-019-0172-y

**Published:** 2019-12-16

**Authors:** Dicky Yulianda, Andy Indra Sati, Akhmad Makhmudi, Gunadi

**Affiliations:** Pediatric Surgery Division, Department of Surgery, Faculty of Medicine, Universitas Gadjah Mada/Dr. Sardjito Hospital, Yogyakarta, 55281 Indonesia

**Keywords:** Complication, Delphi scoring system, Developing country, Hirschsprung diasease, Preoperative Hirschsprung-associated enterocolitis (HAEC)

## Abstract

**Background:**

Hirschsprung-associated enterocolitis (HAEC) is a life-threatening complication of Hirschsprung disease (HSCR), that might occur preoperatively. We investigated the risk factors of preoperative HAEC.

**Method:**

We retrospectively reviewed all medical records of HSCR patients admitted at Dr. Sardjito Hospital, Indonesia from March 2012 until March 2015. Diagnosis of HAEC was determined using the Delphi scoring system.

**Results:**

Sixty-one HSCR patients were involved in this study, of whom 48 were males and 13 females. Eighteen percent (11/61) patients had a preoperative HAEC. The most common findings of the HAEC score found in our patients were distended abdomen (100%) and dilated loops of bowel (100%), followed by lethargy (72.7%), cut-off sign in rectosigmoid with absence of distal air (72.7%), leukocytosis (72.7%), and shift to left (63.6%). There was no association between gender, age of HSCR diagnosis, early/late diagnosis during neonatal period, aganglionosis type, albumin level nor body mass index with preoperative HAEC (*p* = 0.69, 0.76, 0.33, 1.0, 0.86, and 0.50, respectively). In addition, the maternal age, gestational age, and maternal education level also did not correlate with the development of preoperative HAEC (*p* = 0.71, 0.59, and 0.32, respectively).

**Conclusion:**

The incidence of preoperative HAEC in our hospital is considered relatively moderate, with the most common findings of distended abdomen and dilated loops of bowel. None of the identified risk factors have an association with the development of HAEC in our patients.

## Background

Hirschsprung disease (HSCR) is a complex genetic disorder characterized by an absence of ganglion cells in the intestines, resulting in a functional obstruction of the bowels [1]. Its incidence varies among populations ranging from 1:5000 to 1:3250 live-births in European and Indonesian, respectively [1, 2].

Hirschsprung-associated enterocolitis (HAEC) is a life-threatening complication of HSCR [3]. It might occur preoperatively or following pull-through procedure [3–6]. We have shown that the frequency of HAEC following Soave and Duhamel procedures are 10 and 28%, respectively [4], while HAEC occurs preoperatively in 6–60% HSCR patients [3].

There are several risk factors known for preoperative HAEC, however, are still show conflicting findings [3, 5–8]. Therefore, we investigated the risk factors of preoperative HAEC in Indonesia.

## Material and methods

### Subjects

The inclusion criteria of our study were HSCR patients of < 18 years of age who were admitted to Dr. Sardjito Hospital, Indonesia, from March 2012 until March 2015 and underwent a histopathological examination [4, 9], while the exclusion criteria were incomplete medical record. All HSCR patients received a rectal washout while waiting for the surgery.

The Ethical Committee of Faculty of Medicine, Universitas Gadjah Mada/Dr. Sardjito Hospital gave prior approval for this study (KE/FK/713/EC/2015). A written informed consent has been given by the parents’ patients before joining the study.

### HAEC

HAEC was determined using the Delphi scoring system, consisting of 16 criteria including clinical history, physical examination, radiologic and laboratory findings. HAEC is determined when the score is ≥10 [4, 10].

### Statistical analysis

Data are presented as number and percentages for categorical variables. The chi-square or Fisher exact test was used to evaluate the differences of prognostic factors and maternal factors of the preoperative HAEC between groups. IBM SPSS Statistics version 21 (Chicago, USA) was used for statistical analysis.

## Results

### Baseline characteristics

Sixty-one HSCR patients were included in final analysis, involving 48 males and 13 females. Most HSCR patients were diagnosed during the neonate period (54%) and showed short-segment aganglionosis (88.5%) (Table 1).

**Table 1:** Clinical characteristics of HSCR patients admitted to Dr. Sardjito Hospital, Indonesia

Characteristics	n (%)
Gender	
▪ Male	48 (78.7)
▪ Female	13 (21.3)
Age at HSCR diagnosis	
▪ Neonates	33 (54)
√ Early diagnosis (≤7-day-old)	12 (36.4)
√ Late diagnosis (> 7–28-day-old)	21 (63.6)
▪ Post-neonates	28 (46)
HSCR aganglionosis type	
▪ Short-segment	54 (88.5)
▪ Long-segment	7 (11.5)
▪ Total colon aganglionosis	0

*HSCR* Hirschsprung disease

### Preoperative HAEC

Eighteen percent (11/61) of the patients had a preoperative HAEC. The most common findings of the HAEC score found in our patients were distended abdomen (100%) and dilated loops of bowel (100%), followed by lethargy (72.7%), cut-off sign in rectosigmoid with absence of distal air (72.7%), leukocytosis (72.7%), and shift to left (63.6%) (Table 2).

**Table 2:** HAEC scoring system findings in HSCR patients admitted to Dr. Sardjito Hospital, Indonesia

HAEC Score	Preoperative HAEC n (%)
History	
▪ Diarrhea with explosive stool	4/11 (36.4)
▪ Diarrhea with foul-smelling stool	3/11 (27.3)
▪ Diarrhea with bloody stool	1/11 (9.1)
▪ History of enterocolitis	6/11 (54.5)
Physical Examination	
▪ Explosive discharge of gas and stool on rectal examination	6/11 (54.5)
▪ Distended abdomen	11/11 (100)
▪ Decreased peripheral perfusion	2/11 (18.2)
▪ Lethargy	8/11 (72.7)
▪ Fever	6/11 (54.5)
Radiologic Examination	
▪ Multiple air fluid levels	6/11 (54.5)
▪ Dilated loops of bowel	11/11 (100)
▪ Sawtooth appearance with irregular mucosal lining	1 /11 (9.1)
▪ Cutoff sign in rectosigmoid with absence of distal air	8/11 (72.7)
▪ Pneumatosis	1/11 (9.1)
Laboratory Finding	
▪ Leukocytosis	8/11 (72.7)
▪ Shift to left	7/11 (63.6)

*HAEC* Hirschsprung-associated enterocolitis, *HSCR* Hirschsprung disease

### Association of prognostic factors and preoperative HAEC

There was no association between gender, age of HSCR diagnosis, early/late diagnosis during neonatal period, aganglionosis type, albumin level and body mass index with preoperative HAEC (*p* = 0.69, 0.76, 0.33, 1.0, 0.86, and 0.50, respectively) (Table 3).

**Table 3:** Association of prognostic factors and preoperative HAEC in Dr. Sardjito Hospital, Indonesia

Prognostic factor	HAEC (n, %)	Non-HAEC (n, %)	*P*-value*	OR (95% CI)
Gender				
▪ Male	8/11 (72.7)	40/50 (80)	0.69	0.67 (0.15–2.98)
▪ Female	3/11 (27.3)	10/50 (20)		
Age at HSCR diagnosis				
▪ Neonates	5/11 (45.5)	28 (56)	0.76	0.65 (0.18–2.43)
▪ Post-neonates	6/11 (54.5)	22 (44)		
HSCR diagnosis at neonatal period				
▪ Early diagnosis (≤7-day-old)	3/5 (60)	9/28 (32.1)	0.33	3.17 (0.44–22.41)
▪ Late diagnosis (> 7-day-old)	2/5 (40)	19/28 (67.9)		
HSCR aganglionosis type				
▪ Short-segment	10/11 (90.9)	44/50 (88)	1.0	1.36 (0.15–12.63)
▪ Long-segment	1/11 (9.1)	6/50 (12)		
▪ Total colon aganglionosis	0	0		
Albumin level				
▪ Hypoalbuminemia (< 3.5 g/dL)	5/11	24/50 (48)	0.86	0.90 (0.24–3.35)
▪ Normal (≥3.5 g/dL	6/11	26/50 (52)		
Body mass index				
▪ Overweight (>95th percentile or ≥ 25)	4/11	12/50 (24)	0.50	1.81 (0.45–7.26)
▪ Normal (5th – 95th percentile or 18.5 – < 25)	6/11	36/50 (72)		
▪ Underweight (<5th percentile or < 18.5)	1/11	2/50 (4)		

*CI* Confidence interval, *HAEC* Hirschsprung-associated enterocolitis, *HSCR* Hirschsprung disease, *OR* Odds ratio

*significant if *p <* 0.05

### Association of maternal risk factors and preoperative HAEC

In addition, the maternal age, gestational age, and maternal education level also did not correlate with the development of preoperative HAEC (*p* = 0.71, 0.59, and 0.32, respectively) (Table 4).

**Table 4:** Association of maternal risk factors and preoperative HAEC in Dr. Sardjito Hospital, Indonesia

Maternal risk factor	HAEC (n, %)	Non-HAEC (n, %)	*P*-value*	OR (95% CI)
Gestational age				
▪ Preterm	3/11 (27.3)	9/50 (18)	0.59	1.71 (0.38–7.74)
▪ Aterm	8/11 (72.7)	38/50 (76)		
▪ Postterm	0	3/50 (6)		
Maternal age				
▪ ≥35-year-old	4/11 (36.4)	13/50 (26)	0.71	1.63 (0.41–6.47)
▪ < 35-year-old	7/11 (63.6)	37/50 (74)		
Maternal education level				
▪ Low-level	4/11 (36.4)	9/50 (18)	0.32	2.60 (0.62–10.82)
▪ Mid-level	5/11 (45.4)	34/50 (68)		
▪ High-level	2/11 (18.2)	7/50 (14)		

*CI* Confidence interval, *HAEC* Hirschsprung-associated enterocolitis, *HSCR* Hirschsprung disease, *OR* Odds ratio

*significant if *p* < 0.05

## Discussion

Here, we show that the incidence of preoperative HAEC in our institution was relatively moderate (18%). It is similar with previous studies [11–13]. This might be related to the fact that most of our patients were diagnosed during the neonatal period (~ 60%). Previous study revealed that the incidence of HAEC is lower in patients who were diagnosed with HSCR within the first week of life (11%) compared with those infants who were diagnosed with HSCR after the first of week of life (24%) [14]. Another study also presented a similar pattern (early diagnosis: 12% *vs.* late diagnosis: 63%) [15]. Interestingly, the incidence of preoperative HAEC and its mortality was gradually decreased in Japan from previous decade to current one of HSCR patients group (1978–1982 *vs.* 1988–1992 *vs.* 1998–2002: 29.2%/6.5% *vs.* 29.1%/4.9% *vs.* 17.3%/0.7%, respectively). These findings might be due to an earlier diagnosis for the current HSCR patients in Japan [16]. However, our study did not show any difference in HAEC incidence between patients with late diagnosis (2/21, 9.5%) and early diagnosis of HSCR (3/12, 25%) (*p* = 0.33) (Table 3). Our findings are compatible with previous report [17]. Interestingly, the incidence of HAEC was found declining after the neonatal period [5, 12], as a result of the improvement of mucosal defenses or HSCR variants [12].

Diagnosis of HSCR is established according to clinical features, contrast enema, manometry, and histopathology as gold standard [1, 9]. For histopathological staining, hematoxylin and eosin and S100 are the most common staining used in many institutions to assess the presence of ganglion cell and hypertrophic of nerve trunks [9]. Molecular genetic has becoming popular to diagnose an HSCR at molecular level [1]. Most HSCR patients show a variant in *RET* gene [1]. Early definitive diagnosis of HSCR is very important as a key element in further accurate treatment to avoid complications, such as HAEC [18].

Trisomy 21 has been known as a risk factor for HAEC in infants with HSCR [7, 12, 19, 20]. Since the maternal age of ≥35-year-old has a higher risk for having an infant with trisomy 21 [21], we expected older maternal age had more infants with HAEC, however, our findings may support the hypothesis trisomy 21 is not a risk factor for HAEC [5]. Since we retrospectively extracted data from medical records, unfortunately, we do not have a complete data on HAEC infants with trisomy 21.

Only one (1.6%) long-segment patient developed HAEC in our study. We did not find any association between aganglionosis length and HAEC. Previous studies presented that the incidence of HAEC is higher in HSCR long-segment than short-segment patients [22, 23]. This discrepancy might be due to a small sample size in our report. Female gender might increase the risk for HAEC [7], however, the evidence is still lacking [22]. Our study results support the assumption that gender does not affect the incidence of HAEC [22] (Table 3).

There are several intervention methods to prevent HAEC, such as routine washout and probiotics. Our moderate incidence of preoperative HAEC (18%) might relate to our administering routine washout to HSCR patients. Washout procedure decreases fecal stasis and bacterial load, resulting in preventing colonic distension [12]. Regarding probiotics to prevent HAEC, it is not our routine procedure yet, therefore, it is necessary to perform a clinical trial to clarify the role of probiotics in HAEC. How the probiotics can prevent the development HAEC, however, is still controversy [12].

Most HAEC patients showed distended abdomen (100%) and lethargy (72.7%). These clinical findings are considered as “classic” diagnostic criteria [22]. There are several attempts to standardize the diagnostic criteria of HAEC [6, 10]. The current one is developed not only to easily diagnose a HAEC, but also to allow a consistent approach to the HAEC management [3].

Given the fact that hypoalbuminemia has been detected in HAEC *Ednrb*−/− rats [24] and might affect the mortality of HSCR mouse model [25], we hypothesized hypoalbuminemia as one of the risk factors for HAEC. We failed to prove those hypotheses (Table 3).

Since premature infants have a higher risk for having necrotizing enterocolitis [26], we considered premature birth as a risk factor for HAEC. Unfortunately, we did not find any difference in HAEC incidence between premature and aterm/postterm infants. We also speculated that higher educated mothers will show more awareness of early diagnosis of HSCR, thus, preventing HAEC for their infants. However, this hypothesis was not proved.

## Conclusion

The incidence of preoperative HAEC in our hospital is considered relatively moderate, with the most common findings of distended abdomen and dilated loops of the bowel. None of the identified risk factors have an association with the development of HAEC in our patients. It might be due to the small sample size, becoming limitation of our study.

## Data Availability

All data generated or analyzed during this study are included in the submission. The raw data are available from the corresponding author on reasonable request.

## Abbreviations

HAEC: Hirschsprung-associated enterocolitis
HSCR: Hirschsprung disease
